# Depressive symptoms and health-related quality of life in a heterogeneous psychiatric sample: conditional indirect effects of pain severity and interference

**DOI:** 10.1186/s12888-021-03470-1

**Published:** 2021-09-27

**Authors:** Wen Lin Teh, Jianlin Liu, Pratika Satghare, Ellaisha Samari, Yee Ming Mok, Mythily Subramaniam

**Affiliations:** 1 Research Division, Institute of Mental Health, Buangkok Green Medical Park, 10 Buangkok View, Singapore, 539747 Singapore; 2 Department of Mood and Anxiety, Institute of Mental Health, Singapore, 539747 Singapore

**Keywords:** Pain, Depressive symptoms, Health-related quality of life, Psychiatric outpatients

## Abstract

**Background:**

Few studies have examined clinically relevant mechanisms that underlie the association between two important indices of recovery— depression severity and health-related quality of life (HRQOL) in psychiatric outpatients. This study aimed to explicate the roles of pain interference and pain severity as mediating and moderating mechanisms in the relationship between depressive symptoms and HRQOL.

**Methods:**

Data from 290 outpatients diagnosed with schizophrenia (*n* = 102), depressive (*n* = 98), and anxiety (*n* = 90) disorders were examined. Participants completed a set of questionnaires that queried their sociodemographic statuses, current pain severity and interference levels, depression severity levels, and HRQOL. Subsequently, mediation and moderation analyses were conducted.

**Results:**

Analyses revealed that pain interference fully mediated the relationship between depressive symptoms and physical (34% of the total effect) but not mental HRQOL. At high pain levels (+ 1 SD from mean), depressive symptoms may interfere with physical quality of life through pain interference, but this was not present at low pain levels (− 1 SD from mean).

**Conclusions:**

Prolonged pain symptoms could negatively influence psychiatric recovery beyond the physical aspect of HRQOL. These results thus imply a need to detect and manage severe physical pain complaints at the acute stage in psychiatric outpatients.

## Introduction

Symptoms of depression, such as low mood, fatigue, and low self-esteem, can adversely affect recovery in psychiatric illnesses [1, 2]. Depressive symptoms are, however, prevalent, due to the overlapping aetiologies between severe mental illnesses and clinical depression [3, 4] such as in schizophrenia [5] and anxiety disorders [6]. More generally, clinical depression adversely affects all aspects of health-related quality of life (HRQOL); [7–9])─a multidimensional construct that involves subjective evaluations of one’s well-being in various life domains concerning physical and mental health [10]. Unlike other generic measures of quality of life, HRQOL is of greater relevance in healthcare due to its close association with disease recovery [10–12].

The negative effects of depressive symptoms on both overarching domains of HRQOL are well documented, accounting for a modest amount (11–33%) of variance of the physical aspect and a large amount of variance (32–52%) of the mental aspect of HRQOL [13, 14]. A modest amount of variance shared with HRQOL suggests that there are other unmeasured constructs that may further account for its association through potential underlying pathways [15–17]. While there have been attempts to identify the underlying mechanisms between psychiatric symptoms and psychosocial functioning [18, 19], very few attempts have been made to identify clinically relevant mechanisms that are specific to relevant to psychiatric symptoms *and* both physical and mental aspects of HRQOL among patients with psychiatric disorders. Thus, we propose that pain could function as a mediating/moderating mechanism underlying the link between depressive symptoms and HRQOL via its level of severity and interference.

Pain is defined as “an unpleasant sensory and emotional experience associated with, or resembling that associated with, actual or potential tissue damage” [20]. Clinical pain is commonly referred to as pathological physical pain that has a lasting and severe negative impact on health and well-being. While it is typically assumed that pain symptoms are solely relevant to depressive disorders as they are highly comorbid [21], research has shown that this is largely untrue, as physical pain symptoms can occur across severe mental illnesses [22]. According to meta-analytic studies, pain symptoms (including chronic pain) have been reported in 35% of individuals with schizophrenia [23], but more substantially, in 65% of individuals with depression [24]. Pain complaints in primary care patients are known to be associated with greater anxiety symptoms or comorbid anxiety disorders [25, 26].

Depression and clinical pain are inter-connected by neurobiological pathways [27, 28] and can have reciprocal effects on each other; their synergistic effects are reported to have significant impartments on quality of life. For instance, having *both* severe depressive symptoms and clinical pain are associated with greater disability than those with either one condition only [29, 30]. Additionally, pain interference, which is the level of intrusion into daily living that is affected by pain, significantly affects global functioning in severe mental illnesses [22]. In various other studies, pain interference reportedly play a significant mediating role in the association between primary functioning (e.g. affective outcomes, motor or cognitive functioning) and secondary functioning (e.g. self-reported overall/generic functioning and disability) in individuals with various physical health conditions [31–33].

Depression, pain, and quality of life have been shown to be associated in numerous studies, but most were studied in isolation as bivariate conditions and within the boundaries of physical illnesses (as the primary condition). Moreover, while it is well-documented that depressive symptoms underlie pain and functioning in patients primarily diagnosed with chronic physical disorders [34, 35], very little is understood about how current pain could explain the association between depressive symptoms and functioning in patients primarily diagnosed with psychiatric disorders. Thus, the purpose of this study was to examine the role of pain (severity and interference) in the association between depressive symptoms and health-related quality of life (HRQOL) outcomes in a heterogeneous psychiatric sample. We had chosen to study clinical pain in a transdiagnostic sample as pain complaints are prevalent across severe mental illnesses [22]. Furthermore, schizophrenia and affective disorders (i.e., depressive and anxiety disorders) comprise the majority of outpatient cases seen at the Institute of Mental Health (IMH), the sole tertiary psychiatric hospital in Singapore. Thus, the inclusion of these patients in the study ensures that the study results are relevant to the population served by the institution.

**Fig. 1:**
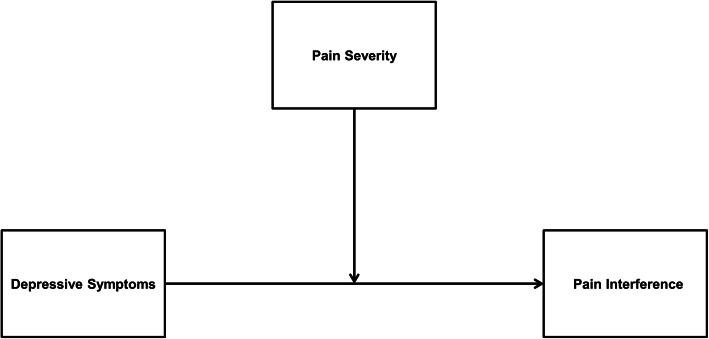
Conceptual illustration of hypothesized moderation model (Model 1). The conceptual illustration depicts the interaction between depressive symptoms and pain severity on pain interference

**Fig. 2:**
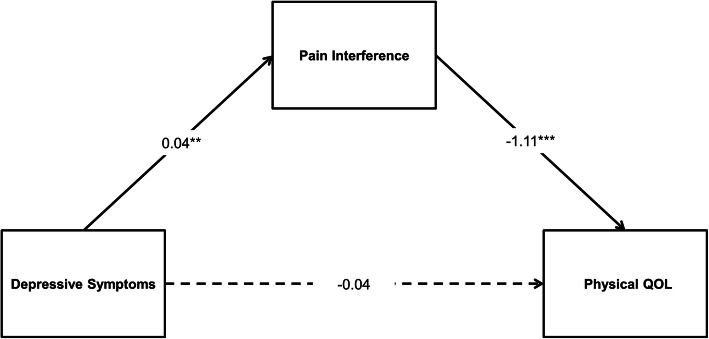
Statistical illustration of hypothesized mediation model (Model 2). Paths depict unstandardized beta coefficients of direct effects of depressive symptoms on physical quality of life (QOL) and pain interference as well as direct effect of pain interference on physical QOL. Solid lines represent significant effects and dotted lines represent non-significant effects. **p < 0.01, ***p < .001

**Fig. 3:**
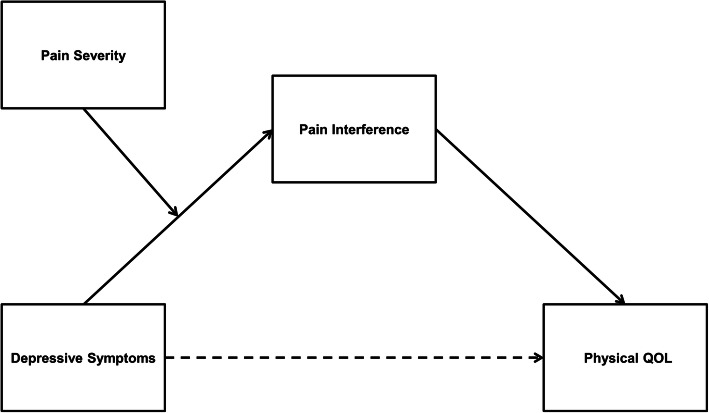
Conceptual illustration of hypothesized moderated mediation model (Model 4). The conceptual illustration depicts the interaction term (i.e., depressive symptoms x pain severity) on pain interference. Paths depict unstandardized beta coefficients of direct effects of depressive symptoms on physical quality of life (QOL) and pain interference as well as direct effect of pain interference on physical QOL. Solid lines represent significant effects and dotted lines represent non-significant effects

We hypothesized that pain severity would moderate the relationship between depressive symptoms and pain interference. Depressive symptoms will be positively associated with pain interference in individuals with high pain severity (hypothesis 1, simple moderation model, Fig. 1). Next, severe pain interference would mediate the relationship between depressive symptoms and HRQOL (hypothesis 2, simple mediation model, Fig. 2). Finally, pain severity would moderate the indirect relationship between depression and HRQOL through pain interference in a moderated mediation model. Specifically, the indirect effect of depressive symptoms on quality of life through pain interference is dependent on high levels of pain severity (hypothesis 3, moderated mediation model, Fig. 3).

## Methods

### Procedure

Individuals seeking outpatient treatment at the Institute of Mental Health (IMH) in Singapore were invited to participate through poster and flyer advertisements posted in the clinic or were referred by their attending doctors. When contacted, members of the research team explained to patients the details of the study which included the risks, benefits, and voluntary nature of their participation. Written informed consent was obtained from all participants after the study procedures had been fully explained. Participants completed a set of questionnaires on their own and were reimbursed an inconvenience fee of 30 Singapore dollars at the end of the survey. The inclusion criteria were: 21 to 65 years of age, literate in the English language, and diagnosed with schizophrenia, depressive, or anxiety disorders. The participants’ diagnoses were extracted from their electronic medical records. Individuals who were deemed unwell or unfit to provide consent by the research team or their attending clinicians at the point of recruitment were excluded. The present study was carried out in accordance with the latest version of the Declaration of Helsinki and was approved by the ethics committee of the Domain Specific Review Board of the National Healthcare Group, Singapore (DSRB No.: 2016/01159).

### Instruments

The measurement tools used in this study are described below:

1. Brief Pain Inventory - Short Form (BPI-SF) is a 9-item questionnaire that measures clinical pain [36]. The items that were utilized in this study are items 3 to 6 which measure pain severity at its “worst”, “least”, “average”, and “now” (current pain i.e., pain experienced in the last 24 h), and 7 items (denoted as A to G) nested within item 9 which measure pain interference or the level of intrusion into daily living (e.g., general activity, mood, or sleep) that is affected by pain. Participants rated each item on a Likert scale of 0 to 10; higher scores indicate greater pain severity and pain interference. Two composite scores measuring two dimensions of pain, 1) pain severity and 2) pain interference, were calculated by taking the average of the sum of 4 (items 3 to 6) and 7 (A to G) items respectively. The BPI-SF has good psychometric properties in assessing pain in primary care [37, 38] and across cultures [39]. Calculated Cronbach alphas showed good internal consistency in our sample, α = 0.94 for pain severity, and α = 0.96 for pain interference.
2. Beck’s Depression Inventory II (BDI-II) is a revised edition of the original BDI scale [40] that measures severity of depressive symptoms. Participants rated each item on a Likert scale of 0 to 3. A total score is calculated by the summation of all 21 items, giving a total score range of 0 to 63, with higher scores representing more severe depressive symptoms. This scale has good psychometric properties [41] and a good level of internal consistency was confirmed in our sample (Cronbach’s α = 0.96).
3. Short Form Health Survey (SF-12) is a short 12-item questionnaire that measures health-related quality of life (HRQOL) by calculating physical (PCS) and mental (MCS) composite summary scores based on Ware et al. [42]. Higher scores on the PCS and MCS denote better quality of life in the respective domains. The SF-12 is a valid measure of HRQOL in severe mental illnesses [43].
4. Sociodemographic questionnaire that had questions on age, gender, ethnicity, and diagnosis.
  Data on age, gender, diagnosis (depression, anxiety, or schizophrenia), and anxiety symptoms measured by Beck’s Anxiety Inventory (BAI) were included in the current study as control variables. These variables were selected based on their significant associations with the primary outcome, such as quality of life [44] and with clinical outcomes, such as depressive symptoms [45, 46].

### Sample size

The data was extracted from a larger study. The sample size was calculated using statistical power calculation for single proportion formula based on prevalence of pain in adult psychiatric patients (18.6%) [47] to produce a precise estimate with a margin of error equal to 5%. We aimed to achieve a target sample of at least 232 patients to provide sufficient precision to measure the prevalence of this condition. After adjusting for 25% incomplete returns, the sample size required to establish prevalence of chronic pain in a psychiatric outpatient population was 300 patients. A final sample of 290 participants were analysed. Mediation and moderation analyses was conducted on a total of 242 participants after listwise deletion of missing values. 

### Data analysis

All statistical analyses were performed using the Statistical Package for Social Sciences (SPSS) version 25.0, with alpha set at 0.05 for all procedures. Individual t-tests, analysis of variance (ANOVA) and correlation analyses were conducted to examine the bivariate associations among all study variables. Mediation and moderation analyses based on ordinary least squares regressions were performed using the PROCESS 3.3 tool [48]. The effect size in terms of f^2^ was calculated and interpreted according to Cohen’s (1988) criteria: f^2^ = 0.2 (small), f^2^ = 0.25 (moderate), and f^2^ = 0.4 (large).

The hypothesized moderation model (Model 1; Fig. 1) examined whether pain severity moderated the relationship between depressive symptoms and pain interference while controlling for age, gender, primary diagnosis, and anxiety symptoms. Variables depressive symptoms and pain severity were first centered and multiplied to obtain the interaction term (depressive symptoms x pain severity). Next, moderation analysis was conducted using depressive symptoms, pain severity, the interaction term as the independent variables, and pain interference as the dependent variable. Significant interactions were probed using the pick-a-point approach (±1 standard deviation; [49]).

Two hypothesized mediation models controlling for the same set of variables were examined to determine whether pain interference mediated the relationship between depressive symptoms and HRQOL (Model 2; Fig. 2) and whether pain interference mediated the relationship between depressive symptoms and mental quality of life (Model 3). Bootstrapped 95% confidence intervals (CI) based on 5000 iterations were used to determine the significance of indirect effects [48].

Following our results of Models 1 to 3, we tested the hypothesis that pain severity moderated the indirect effect of depressive symptoms and physical quality of life through pain interference while controlling for the same set of variables (Model 4; Fig. 3). This conditional process model of moderated mediation simultaneously examined the integrated influence of pain severity (moderator) and pain interference (mediator) on the relationship between depressive symptoms and physical quality of life. In our hypothesized moderation mediation model, we tested the moderation of the effect of depressive symptoms on pain interference. The moderation of the indirect effect was determined by the statistical significance of the index of moderated mediation [48]. Finally, the analyses were repeated by replacing the composite score of pain severity with a single item score on the BPI-SF for “average” pain, and the results were similar.

## Results

### Sample characteristics and preliminary analyses

The mean age of participants was 40 years (M = 39.6, SD = 11.6) and the sample comprised three diagnostic groups: schizophrenia (35.2%), depression (33.8%), and anxiety disorders (31%). Slightly more than half of the sample was female (51%). ANOVA analyses revealed significant differences in BDI-II scores, F(2,249) =23.3, *p* < .001, and MCS scores, F(2,285) =27.2, *p* < .001, between diagnostic categories. T-tests indicated no significant differences in BDI-II, BPI-SF, PCS, and MCS scores between gender. See Tables 1 and 2 for a summary of the descriptive characteristics and correlations.

**Table 1:** Means, standard deviations, and 95% confidence intervals of BDI-II, BPI-SF, and SF-12 by diagnosis categories and gender

	BDI-II			BPI-SF						SF-12					
				Pain severity			Pain interference			PCS			MCS		
	Mean (SD)	95% CI		Mean (SD)	95% CI		Mean (SD)	95% CI		Mean (SD)	95% CI		Mean (SD)	95% CI	
		lower	upper		lower	upper		lower	upper		lower	upper		lower	upper
Diagnosis categories															
Schizophrenia (n = 102)	11.7(12.5)	9.0	14.3	2.4(2.5)	1.9	2.9	2.3(2.5)	1.8	2.8	46.7(8.3)	44.9	48.4	45.7(10.8)	43.5	48.0
Depression (n = 98)	26.9(16.7)	23.2	30.6	3.1(2.7)	2.5	3.7	3.2(3.1)	2.5	3.9	47.1(9.3)	45.0	49.1	34.0(12.5)	31.3	36.8
Anxiety (n = 90)	20.8(15.6)	17.2	24.4	2.7(2.2)	2.2	3.2	2.4(2.6)	1.8	3.0	48.6(8.6)	46.6	50.5	37.8(11.9)	35.1	40.5
Gender															
Male (*n* = 142)	20.0(16.1)	17.1	22.9	2.8(2.6)	2.3	3.3	2.8(2.8)	2.3	3.3	47.9(8.8)	46.3	49.4	39.4(12.8)	37.1	41.7
Female (*n* = 148)	19.0(16.3)	16.1	22.0	2.6(2.5)	2.2	3.1	2.4(2.7)	2.0	2.9	47(8.6)	45.4	48.5	39.4(12.6)	37.2	41.7

note: BDI-II is the Beck’s Depression Inventory II; BPI-SF is the Brief Pain Inventory Short Form Scale; SF-12 is the Short Form Health Survey comprising of the Physical and Mental composite summary scores PCS and MCS; SD is standard deviation; CI is confidence interval; Significant differences were found between diagnosis categories in BDI-II and MCS scores *p* < .001; No significant differences between gender were present in this sample; Missing values were deleted listwise

**Table 2:** Bivariate correlations between age, BDI-II, BPI-SF, SF-12 scores

	1	2	3	4	5	6
age	–	**−0.18**	0.11	0.07	**−0.32**	**0.25**
BDI-II		–	**0.42**	**0.52**	**−0.28**	**− 0.76**
Pain severity			–	**0.82**	**−0.51**	**−0.29**
Pain interference				–	**−0.53**	**−0.36**
PCS					–	−0.003
MCS						–

note: BDI-II is the Beck’s Depression Inventory II; Pain severity and interference are components of BPI-SF; Physical and Mental composite summary scores PCS and MCS are components of the SF-12; values in bold represent significance at *p < .*05

### Moderation analyses

**Fig. 4:**
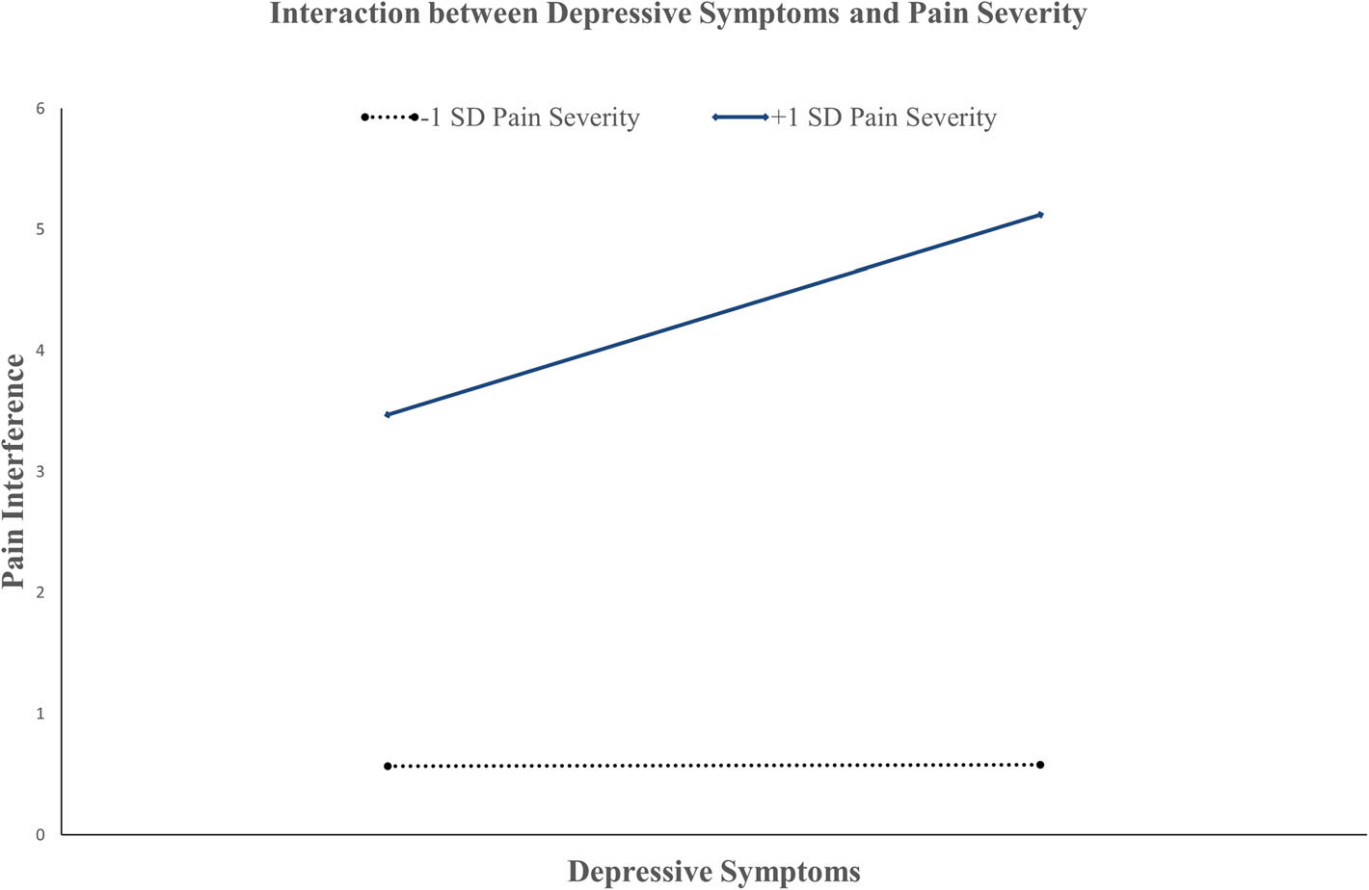
Results illustrating the interaction between depressive symptoms and pain severity on pain interference. Values plotted were based on predicted pain interference scores from the regression equation using centered mean and centered scores ±1 standard deviation (SD) from the mean of depressive symptoms and pain severity

Moderation analysis of Model 1 revealed a significant positive interaction between depressive symptoms and pain severity on pain interference (f^2^ = 2.72, *p* < .001): depressive symptoms x pain severity (b = 0.01, SE = 0.002, 95% CI [0.005 to 0.01], *p* < .001). Figure 4 depicts the interaction plot of depressive symptoms and pain severity on pain interference. The interaction revealed that at high pain severity (+ 1 SD from mean; conditional effect = 0.05, SE = 0.01, 95% CI [0.03 to 0.07], *p* < .001), depressive symptoms were significantly related to more severe pain interference. Depressive symptoms were not significantly related to pain interference at low pain severity (− 1 SD from mean; conditional effect = 0.0003, SE = 0.01, 95% CI [− 0.02 to 0.02], *p* = 0.98). The results suggest that the association between depressive symptoms and pain interference was observed only in individuals with high pain severity.

### Mediation analyses

Mediation analysis of Model 2 revealed a significant indirect effect of depressive symptoms on physical quality of life via pain interference, b = − 0.04, *SE* = 0.02, 95% CI [− 0.08 to − 0.01]. The ratio of the indirect effect (i.e., the percentage of total effect that is accounted for and by the indirect effect) was 54.6%. The direct effect of depressive symptoms on physical quality of life was not significant, b = − 0.04, *SE* = 0.04, 95% CI [− 0.12 to 0.05]. This suggests that pain interference fully mediated the effects of depressive symptoms on physical quality of life (Fig. 2). The mediation model (Model 2) accounted for 34% of the variance in physical quality of life and achieved a large effect size (f^2^ = 0.52). In contrast to Model 2, mediation analysis of Model 3 revealed a non-significant indirect effect of depressive symptoms on mental quality of life via pain interference, b = − 0.004, *SE* = 0.01, 95% CI [− 0.03 to 0.02]. Therefore, pain interference did not mediate the effects of depressive symptoms on mental quality of life.

### Moderated mediation analysis

Following our significant results for Models 1 and 2, we integrated both models together and analysed a conditional process model of moderated mediation (Model 4; Fig. 3). The results revealed a significant index of moderated mediation (Index = − 0.01, *SE* = 0.004, 95% CI [− 0.02 to − 0.01]. This indicates that the indirect effect of depressive symptoms on physical quality of life via pain interference was dependent on the level of pain severity (f^2^ = 0.52). Specifically, at high levels (+ 1 SD from mean) of pain severity, the indirect effect was significant (b = − 0.06, *SE* = 0.02, 95% CI [− 0.10 to − 0.03]); however, at low levels (− 1 SD from mean) of pain severity, the indirect effect was reduced and rendered non-significant. The effect size of the moderated mediation (i.e., the ratio of the conditional indirect effect to total effect) was 61.3% for high pain severity.

## Discussion

Past research found that psychiatric outpatients with depressive symptoms generally report lower health-related quality of life [7, 50] and it was proposed that pain could function as a mechanism underlying this link, via its severity and its level of interference. Specifically, this study found that pain interference fully mediated the relationship between depressive symptoms and physical (explaining a large variance, 34% of the total effect) but not mental HRQOL (Model 2 but not 3 was supported), and the indirect effect of pain interference was contingent on pain severity. At high pain levels (+ 1 SD from mean), depressive symptoms may interfere with physical quality of life through pain interference, but this was not present at low pain levels (− 1 SD from mean).

Pain interference could indicate the presence of physical health complaints [22], comorbid physical conditions [51, 52], and/or medically unexplained physical symptoms [53] in the study sample. Psychosomatic mechanisms, such as a hypersensitivity to or a catastrophic interpretation of physical pain sensations [54], may amplify pain interferences on day-to-day living and overall physical HRQOL during heightened depressive states. It is further evidenced in a study by Osborne et al. (2007), that the intensity of pain greatly influences the level of interference on day-to-day functioning, accounting for 35% of unique variance of pain interference [55]. Coupled with severe pain, the indirect pathway of pain interference on the association between depressive symptoms and physical HRQOL becomes amplified.

Current pain interference did not mediate the association between depressive symptoms and mental QOL which was against expectations; however, several reasons could account for it. It is well established that pain is the strongest predictor of physical QOL and depressive symptoms are the strongest predictor of mental QOL [56]. In this study, depressive symptoms overlapped significantly with mental HRQOL; bivariate correlation analyses showed that pain interference was moderately correlated with mental HRQOL (MCS; *r* = − 0.36) and by contrast, depressive symptoms (BDI-II) were highly correlated with MCS (*r* = 0.76) due to highly overlapping psychological constructs. Chronic pain is associated with numerous mental health related outcomes, such as future onset of depression [57], insomnia symptoms [58], fatigue [59], and loneliness [60] or withdrawal from social relationships [61]. As compared to current levels of pain which was the focus of the current study, pain could have a more deleterious impact on mental health and functioning if it turns chronic over time [62]. Thus, our findings may differ from studies, such as by Wong et al. [63], due to the focus on current and not chronic pain. Thus, the present evidence suggests the need to address acute physical pain complaints as theye have the potential to impact on mental well-being if left unaddressed for too long.

As the current results are based on cross-sectional data, there are several limitations that need to be considered. First, due to the complex and bi-directional nature of the variables investigated, we are unable to ascertain causal links between the variables despite utilizing mediation analyses which could provide evidence for temporal relationships. Second, there is also a lack of objective measurements to corroborate self-reported pain, which could aid in differentiating physical from psychosomatic pain. Third, comorbid physical conditions were neither controlled for nor analysed in this study. Our results are considered preliminary and further longitudinal work is required. Finally, while our sample comprised of a diverse group of outpatients that are commonly seen in the outpatient clinics, the generalizability of our results is limited to those who were well and willing to participate.

Detecting and addressing severe pain complaints in psychiatric outpatients should be endorsed in recovery. However, individuals seeking help at psychiatric hospitals generally present more urgent psychological issues at hand and pain becomes a secondary concern. As a secondary symptom, pain is under-detected and undertreated due to a lack of screening [64]. Issues such as these highlight the challenges of treating the clinical complexities of physical-mental comorbidities in psychiatry [65, 66]. Albeit preliminary, our results may be supportive of interventions that address pain in recovery, especially those presenting with severe depressive symptoms and pain complaints at the outpatient clinics. For instance, supplementing pain self-management courses into existing treatments and interventions, such as pharmacotherapy or cognitive behavioural therapy, could be considered as they have shown promise at improving psychological, functioning, and quality of life outcomes over time [67–69].

This study tested the moderating and mediating roles of pain severity and interference. More importantly, it addressed important questions about *how* and *when* depressive symptoms would be associated with *which aspect* of quality of life. As a common condition of day-to-day living, pain can vary from mild to severe in intensity. Future longitudinal studies may extend our findings to determine if severe pain interference is an underlying mechanism through which depressive symptoms negatively influence health-related physical quality of life.

## Data Availability

Not applicable.

## Abbreviations

QOL: Quality of Life
HRQOL: Health-related Quality of Life
IMH: Institute of Mental Health
BPI-SF: Brief Pain Inventory - Short Form
BDI-II: Beck’s Depression Inventory II
SF-12: Short Form Health Survey
PCS: physical composite summary score of the SF-12
MCS: mental composite summary score of the SF-12
ANOVA: Analysis of variance

## References

[1] Judd LL, Akiskal HS, Maser JD, Zeller PJ, Endicott J, Coryell W, Paulus MP, Kunovac JL, Leon AC, Mueller TI, Rice JA, Keller MB (1998). Major depressive disorder: a prospective study of residual subthreshold depressive symptoms as predictor of rapid relapse. J Affect Disord.

[2] Buckley PF, Miller BJ, Lehrer DS, Castle DJ (2009). Psychiatric comorbidities and schizophrenia. Schizophr Bull.

[3] Upthegrove R, Marwaha S, Birchwood M (2017). Depression and schizophrenia: cause, consequence, or trans-diagnostic issue?. Schizophr Bull.

[4] Groen RN, Ryan O, Wigman JTW, Riese H, Penninx BWJH, Giltay EJ, Wichers M, Hartman CA (2020). Comorbidity between depression and anxiety: assessing the role of bridge mental states in dynamic psychological networks. BMC Med.

[5] an der Heiden W, Leber A, Häfner H. Negative symptoms and their association with depressive symptoms in the long-term course of schizophrenia. Eur Arch Psychiatry Clin Neurosci 2016;266(5):387–396, 10.1007/s00406-016-0697-2. 10.1007/s00406-016-0697-2 27107764

[6] Cummings CM, Caporino NE, Kendall PC (2014). Comorbidity of anxiety and depression in children and adolescents: 20 years after. Psychol Bull.

[7] Berlim MT, Fleck MPA. Quality of life and major depression. In: Quality of life impairment in schizophrenia, mood and anxiety disorders. Springer; 2007. p. 241–252, 10.1007/978-1-4020-5779-3_12.

[8] IsHak WW, Greenberg JM, Balayan K, Kapitanski N, Jeffrey J, Fathy H (2011). Quality of life: the ultimate outcome measure of interventions in major depressive disorder. Harv Rev Psychiatry.

[9] Karow A, Wittmann L, Schöttle D, Schaefer I, Lambert M (2014). The assessment of quality of life in clinical practice in patients with schizophrenia. Dialogues Clin Neurosci.

[10] Revicki DA, Kleinman L, Cella D (2014). A history of health-related quality of life outcomes in psychiatry. Dialogues Clin Neurosci.

[11] Chen H-Y, Baumgardner DJ, Rice JP. Peer reviewed: Health-related quality of life among adults with multiple chronic conditions in the united states, behavioral risk factor surveillance system, 2007. Prev Chronic Dis. 2011;8(1). PMC3044020 21159221

[12] Carod-Artal FJ, Egido JA (2009). Quality of life after stroke: the importance of a good recovery. Cerebrovasc Dis.

[13] Adan A, Marquez-Arrico JE, Gilchrist G (2017). Comparison of health-related quality of life among men with different co-existing severe mental disorders in treatment for substance use. Health Qual Life Outcomes.

[14] Felker B, Katon W, Hedrick SC, Rasmussen J, McKnight K, McDonnell MB (2001). The association between depressive symptoms and health status in patients with chronic pulmonary disease. Gen Hosp Psychiatry.

[15] Liu J, Abdin E, Verma S, Sim K, Chong SA, Subramaniam M (2019). Clarifying pathways to poor psychological health: the mediating role of psychosocial factors in the relationship between general psychopathology and quality of life impairment in patients diagnosed with schizophrenia. J Clin Psychol.

[16] de Pinho LMG, Pereira AMS, Chaves C, Batista P (2018). Quality of life scale and symptomatology of schizophrenic patients–a systematic review. Eur J Psychiatry.

[17] Eack SM, Newhill CE (2007). Psychiatric symptoms and quality of life in schizophrenia: a meta-analysis. Schizophr Bull.

[18] Buist-Bouwman MA, Ormel J, De Graaf R, De Jonge P, Van Sonderen E, Alonso J (2008). Mediators of the association between depression and role functioning. Acta Psychiatr Scand.

[19] Knight MJ, Baune BT (2018). Executive function and spatial cognition mediate psychosocial dysfunction in major depressive disorder. Front psychiatry.

[20] Raja SN, Carr DB, Cohen M, Finnerup NB, Flor H, Gibson S, Keefe FJ, Mogil JS, Ringkamp M, Sluka KA, Song XJ, Stevens B, Sullivan MD, Tutelman PR, Ushida T, Vader K (2020). The revised International Association for the Study of Pain definition of pain: concepts, challenges, and compromises. Pain..

[21] Kirmayer LJ, Robbins JM, Dworkind M, Yaffe MJ (1993). Somatization and the recognition of depression and anxiety in primary care. Am J Psychiatry.

[22] Abplanalp SJ, Mueser KT, Fulford D (2020). The role of physical pain in global functioning of people with serious mental illness. Schizophr Res.

[23] Stubbs B, Mitchell AJ, De Hert M, Correll CU, Soundy A, Stroobants M (2014). The prevalence and moderators of clinical pain in people with schizophrenia: a systematic review and large scale meta-analysis. Schizophr Res.

[24] Bair MJ, Robinson RL, Katon W, Kroenke K (2003). Depression and pain comorbidity: a literature review. Arch Intern Med.

[25] Means-Christensen AJ, Roy-Byrne PP, Sherbourne CD, Craske MG, Stein MB (2008). Relationships among pain, anxiety, and depression in primary care. Depress Anxiety.

[26] Tsang A, Von Korff M, Lee S, Alonso J, Karam E, Angermeyer MC (2008). Common chronic pain conditions in developed and developing countries: gender and age differences and comorbidity with depression-anxiety disorders. J Pain.

[27] Boakye PA, Olechowski C, Rashiq S, Verrier MJ, Kerr B, Witmans M, Baker G, Joyce A, Dick BD (2016). A critical review of neurobiological factors involved in the interactions between chronic pain, depression, and sleep disruption. Clin J Pain.

[28] Haleem DJ (2019). Targeting Serotonin1A receptors for treating chronic pain and depression. Curr Neuropharmacol.

[29] IsHak WW, Wen RY, Naghdechi L, Vanle B, Dang J, Knosp M (2018). Pain and depression: a systematic review. Harv Rev Psychiatry..

[30] Tsuji T, Matsudaira K, Sato H, Vietri J (2016). The impact of depression among chronic low back pain patients in Japan. BMC Musculoskelet Disord.

[31] Pelletier R, Bourbonnais D, Higgins J, Mireault M, Harris PG, Danino MA (2020). Pain interference may be an important link between pain severity, impairment, and self-reported disability in participants with wrist/hand pain. J Hand Ther.

[32] Varni JW, Nutakki K, Swigonski NL (2020). Cognitive functioning and pain interference mediate pain predictive effects on health-related quality of life in pediatric patients with Neurofibromatosis type 1. Eur J Paediatr Neurol.

[33] Krause JS, Brotherton SS, Morrisette DC, Newman SD, Karakostas TE (2007). Does pain interference mediate the relationship of independence in ambulation with depressive symptoms after spinal cord injury?. Rehabil Psychol.

[34] Ross C, Juraskova I, Lee H, Parkitny L, Stanton TR, Moseley GL, McAuley JH (2015). Psychological distress mediates the relationship between pain and disability in hand or wrist fractures. J Pain.

[35] Hall AM, Kamper SJ, Maher CG, Latimer J, Ferreira ML, Nicholas MK (2011). Symptoms of depression and stress mediate the effect of pain on disability. Pain..

[36] Cleeland CS, Ryan KM. Pain assessment: global use of the brief pain inventory. Ann Acad Med Singap. 1994. 8080219

[37] Tan G, Jensen MP, Thornby JI, Shanti BF (2004). Validation of the brief pain inventory for chronic nonmalignant pain. J Pain.

[38] Cleeland CS, Ryan KM. The brief pain inventory. Pain Res Gr. 1991:143–7.

[39] Cleeland CS, Nakamura Y, Mendoza TR, Edwards KR, Douglas J, Serlin RC (1996). Dimensions of the impact of cancer pain in a four country sample: new information from multidimensional scaling. Pain.

[40] Beck AT, Steer RA, Brown G. Beck depression inventory–II. Psychol Assess. 1996

[41] Dozois DJA, Dobson KS, Ahnberg JL (1998). A psychometric evaluation of the Beck depression inventory–II. Psychol Assess.

[42] Ware JE, Kosinski M, Keller SD (1996). A 12-item short-form health survey: construction of scales and preliminary tests of reliability and validity. Med Care.

[43] Salyers MP, Bosworth HB, Swanson JW, Lamb-Pagone J, Osher FC (2000). Reliability and validity of the SF-12 health survey among people with severe mental illness. Med Care.

[44] Michel G, Bisegger C, Fuhr DC, Abel T (2009). Age and gender differences in health-related quality of life of children and adolescents in Europe: a multilevel analysis. Qual Life Res.

[45] Stavrakaki C, Vargo B (1986). The relationship of anxiety and depression: a review of the literature. Br J Psychiatry.

[46] Piccinelli M, Wilkinson G (2000). Gender differences in depression: critical review. Br J Psychiatry.

[47] Chaturvedi SK (1987). Prevalence of chronic pain in psychiatric patients. Pain..

[48] Hayes AF (2017). Introduction to mediation, moderation, and conditional process analysis: a regression-based approach.

[49] Preacher KJ, Curran PJ, Bauer DJ (2006). Computational tools for probing interactions in multiple linear regression, multilevel modeling, and latent curve analysis. J Educ Behav Stat.

[50] Bonicatto SC, Dew MA, Zaratiegui R, Lorenzo L, Pecina P (2001). Adult outpatients with depression: worse quality of life than in other chronic medical diseases in Argentina. Soc Sci Med.

[51] De Hert M, Correll CU, Bobes J, Cetkovich-Bakmas M, Cohen DAN, Asai I, et al. Physical illness in patients with severe mental disorders. I. Prevalence, impact of medications and disparities in health care. World Psychiatry. 2011;10(1):52. 10.1002/j.2051-5545.2011.tb00014.x PMC3048500 21379357

[52] Teh WL, Cetty L, Jeyagurunathan A, Devi F, Roystonn K, Tang C, Verma S, Subramaniam M (2021). Comorbid physical illnesses in adult outpatients with psychotic disorders: risk factors, psychological functioning, and quality of life outcomes. Soc Psychiatry Psychiatr Epidemiol.

[53] Henningsen P, Zimmermann T, Sattel H (2003). Medically unexplained physical symptoms, anxiety, and depression: a meta-analytic review. Psychosom Med.

[54] Rief W, Broadbent E (2007). Explaining medically unexplained symptoms-models and mechanisms. Clin Psychol Rev.

[55] Osborne TL, Jensen MP, Ehde DM, Hanley MA, Kraft G (2007). Psychosocial factors associated with pain intensity, pain-related interference, and psychological functioning in persons with multiple sclerosis and pain. Pain..

[56] Galvez-Sánchez CM, Montoro CI, Duschek S, Del Paso GAR (2020). Depression and trait-anxiety mediate the influence of clinical pain on health-related quality of life in fibromyalgia. J Affect Disord.

[57] McBeth J, Macfarlane GJ, Silman AJ (2002). Does chronic pain predict future psychological distress?. Pain..

[58] Sit RWS, Yip BHK, Wang B, Chan DCC, Zhang D, Wong SYS. Chronic musculoskeletal pain prospectively predicts insomnia in older people, not moderated by age, gender or co-morbid illnesses. Sci Rep 2021;11(1):1–7, 1593, 10.1038/s41598-021-81390-6. 10.1038/s41598-021-81390-6 PMC7810727 33452447

[59] Lenaert B, Meulders A, van Heugten CM (2018). Tired of pain or painfully tired? A reciprocal relationship between chronic pain and fatigue. Pain..

[60] Smith TO, Dainty JR, Williamson E, Martin KR (2019). Association between musculoskeletal pain with social isolation and loneliness: analysis of the English longitudinal study of ageing. Br J Pain.

[61] Sturgeon JA, Zautra AJ, Arewasikporn A. A multilevel structural equation modeling analysis of vulnerabilities and resilience resources influencing affective adaptation to chronic pain. Pain®. 2014;155(2):292–8. 10.1016/j.pain.2013.10.007 PMC3947000 24120460

[62] Wade JB, Dougherty LM, Archer CR, Price DD (1996). Assessing the stages of pain processing: a multivariate analytical approach. Pain.

[63] Wong WS, Chan STM, Fung VBK, Fielding R (2010). The differential mediating effects of pain and depression on the physical and mental dimension of quality of life in Hong Kong Chinese adults. Health Qual Life Outcomes.

[64] Marazziti D, Mungai F, Vivarelli L, Presta S, Dell’Osso B (2006). Pain and psychiatry: a critical analysis and pharmacological review. Clin Pract Epidemiol Ment Heal.

[65] Mezzich JE, Salloum IM (2008). Clinical complexity and person-centered integrative diagnosis. World Psychiatry.

[66] Sartorius N. Comorbidity of mental and physical disorders: a key problem for medicine in the 21st century. Wiley Online Library; 2018. 10.1111/acps.12888 29637546

[67] Kroenke K, Baye F, Lourens SG, Evans E, Weitlauf S, McCalley S, Porter B, Matthias MS, Bair MJ (2019). Automated self-management (ASM) vs. ASM-enhanced collaborative care for chronic pain and mood symptoms: the CAMMPS randomized clinical trial. J Gen Intern Med.

[68] Nicholas MK, Asghari A, Corbett M, Smeets RJEM, Wood BM, Overton S, Perry C, Tonkin LE, Beeston L (2012). Is adherence to pain self-management strategies associated with improved pain, depression and disability in those with disabling chronic pain?. Eur J Pain.

[69] Dworkin RH, Gitlin MJ (1991). Clinical aspects of depression in chronic pain patients. Clin J Pain.

